# Thermal Transition Properties of Hoki (*Macruronus novaezelandiae*) and Ling (*Genypterus blacodes*) Skin Collagens: Implications for Processing

**DOI:** 10.3390/md9071176

**Published:** 2011-06-28

**Authors:** Kathleen Anne Hofman, Marcus Newberry

**Affiliations:** 1 Seafood Research Unit, The New Zealand Institute for Plant & Food Research Limited, P.O. Box 5114, Port Nelson, 7043, New Zealand; 2 CSIRO Plant Industry, GPO Box 1600, Canberra ACT 2601, Australia; E-Mail: Marcus.Newberry@csiro.au

**Keywords:** collagen, cold water fish, processing waste, thermal denaturation temperature, Rapid Visco™ Analyzer

## Abstract

Hoki (*Macruronus novaezelandiae*) and ling (*Genypterus blacodes*) are cold-water fish caught in New Zealand waters. Their skins are a major component of the post-processing waste stream. Valuable products could be developed from the skins, as they are primarily composed of collagen, which has many commercial applications. We prepared acid soluble collagens (ASC) from hoki and ling skins, and analyzed their thermal denaturation properties using a Rapid Visco™ Analyzer. At slower heating rates the denaturation temperature (TD) of hoki and ling collagens decreased. This result is consistent with the model of irreversible rate kinetics for the denaturation of collagen. We determined the effects of solvents that disrupt hydrogen bonding on ASC stability. Increasing concentrations of urea from 0.1 M to 1.0 M and acetic acid from 0.1 M to 0.5 M decreased TD. This resulted from the effects of these reagents on the hydrogen bonds that stabilize the collagen triple helix.

## 1. Introduction

Hoki and ling are cold water fish species from the Merlucciidae and Ophidiidae families, respectively [1]. Since only approximately 40% of the fish catch is processed into fillets, there are strong incentives to investigate the biochemical nature of the by-products, and to separate out specific molecular fractions for use in high-value applications such as in the food, cosmetic, and nutraceutical industries. In the food industry collagen can function as a gel forming agent, a texturant, as a component of microencapsulated particles to deliver active ingredients, as well as providing the base for edible films and coatings. The skin collagens from cold water marine fish species differ from that sourced from mammals, as they are more acid-soluble, and have low imino acid contents [2]. Because of their acid solubility, these skin collagens readily dissociate into intact collagen molecules, and thus provide a different raw material for potential product applications. Similarly, the low imino acid content affects various functional properties, such as Bloom strength of manufactured gelatins and stability of the triple helix in applications involving whole molecules.

All collagenous proteins have domains with a triple-helical conformation formed by three subunits, or α chains, which have three distinct regions. The largest and middle portion of each α chain contains long sequences of repeating tripeptides, based on the general structure Gly-X-Y, where X is commonly proline and Y is often the modified imino acid hydroxyproline [3]. The triple helix is well stabilized by the internal non-covalent bonding structure such that, at low temperatures, it is highly resistant to proteolytic attack [4]. Collagen triple helix denaturation involves rupture of hydrogen bonds and a rearrangement of the triple helix into a random chain configuration [5]. Fiber-forming collagens in dilute solution show highly co-operative helix-coil transitions at temperatures that are remarkably close to the body temperature of the animal from which the collagen was extracted [6]. This close correlation is found across animal Phyla and the transition temperatures, which range from 5 to 40 °C, are dependent on primary structure, especially the concentration of hydroxyproline residues [2].

For many years, the accepted model of collagen denaturation had been that it was an equilibrium process involving the rupture of hydrogen bonds. However, Miles and coworkers [7] proposed that the characteristic sharp melting point of collagen molecules is governed by irreversible rate kinetics, rather than equilibrium rate kinetics. The irreversible rate kinetics model includes the concept of domains of variable thermal stability along the length of the molecule. Their model stresses the importance of hydroxyproline in stabilizing the triple helix and supports the concept of hydrogen-bonded water-bridges [8,9].

The aim of this study was to understand the thermal stability of ASC extracted from the skins of two cold-water fish species. This was achieved by evaluating the relationship between temperature and collagen denaturation, and by determining the effects of solvents that disrupt hydrogen bonding on the denaturation temperature (*T*_D_). Understanding collagen thermal stability is important in manufacturing from the processing of raw materials to the storage of products. The results of this study are applicable for processing of these skins into functional products that can be used in cosmetics, foods, and nutraceuticals.

## 2. Results and Discussion

### 2.1. Molecular Profile of Prepared Collagens

**Figure 1:**
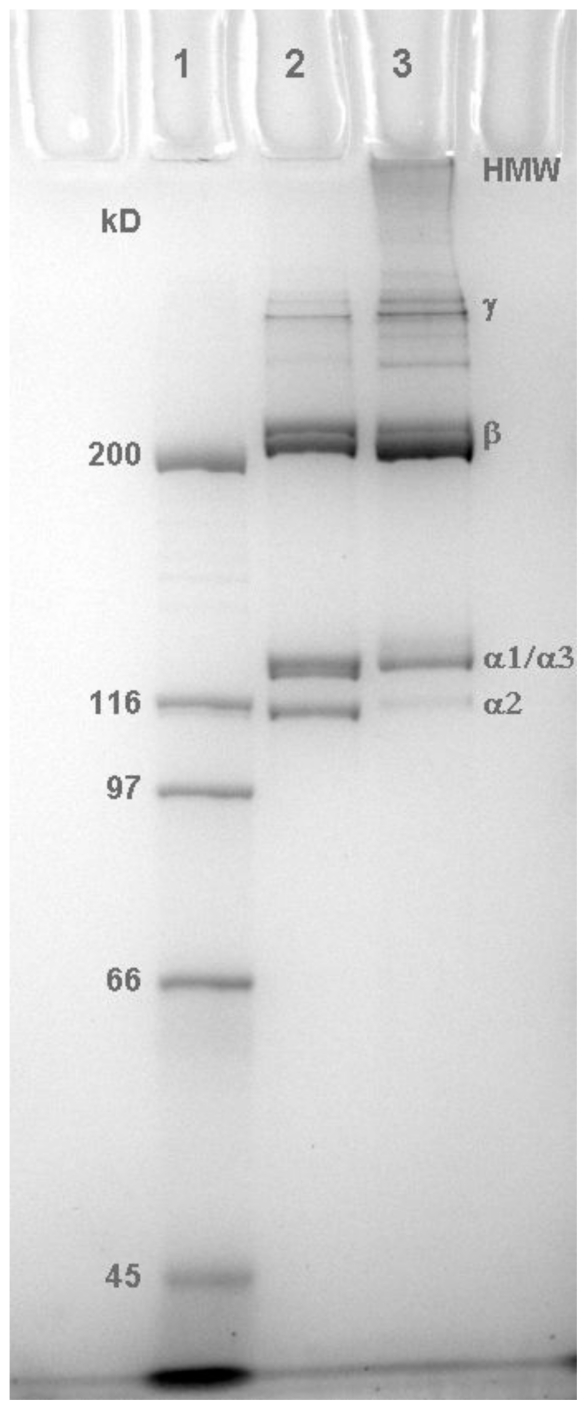
SDS-PAGE (7.5% acrylamide) analysis of hoki and ling collagen preparations. Lane 1: Broad Range marker proteins (Bio Rad™): myosin (200 kDa), β-galactosidase (116 kDa), phosphorylase b (97 kDa), serum albumin (66 kDa) and ovalbumin (45 kDa); Lane 2: hoki skin collagen (1.5 μg); and lane 3: ling skin collagen (2.5 μg).

During the preparation of acid-soluble collagen from the fish skins the acid-labile, inter-molecular lysine derived covalent crosslinks are cleaved to release the collagen. The collagens prepared from hoki and ling were examined by SDS-PAGE (Figure 1).

As shown in Figure 1, the hoki collagen preparation (lane 2) contained α chain subunits and β and γ complexes, as expected under the denaturing conditions of SDS-PAGE. In contrast, the lanes containing the ling collagen preparation showed high molecular weight (HMW) material at the top of the gel, as well as the characteristic α chain subunits and β and γ complexes. The recovery of the denatured β, γ, and HMW components in the gel results from the presence of some interchain covalent crosslinks that were not cleaved during extraction of the collagen in acetic acid. This result indicates that the ling collagen mixture retained some microfibrillar structure. Therefore, the thermal denaturation results for the hoki collagen reflect disruption of the H-bonding stabilizing the molecular helix, whereas those for the ling collagen reflect disruption of the helix as well as the associations that maintain the microfibrils in solution.

### 2.2. Thermal Denaturation Temperature Measurements

**Figure 2:**
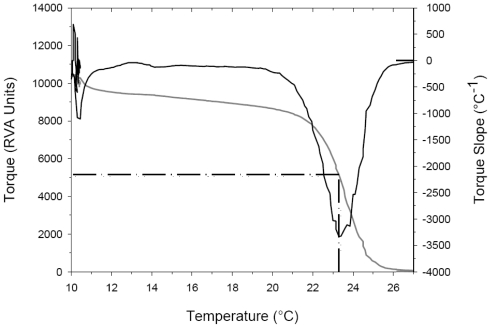
Typical RVA torque (viscosity) curve (gray line) for a sample of hoki collagen in water during heating from 10 to 28 °C at 2 °C/min and corresponding slope of the torque-temperature curve. The minimum in the slope is used to define the denaturation temperature (dashed line). For this sample the denaturation temperature was determined to be 23.3 °C.

When heated in solutions, collagen molecules denature over a narrow range of temperatures, the mid-point of which is referred to as the denaturation temperature (*T*_D_). When monitored as a change in viscosity, *T*_D_ is normally defined as the temperature at which the viscosity has decreased to half of its initial value. One difficulty with this approach is consistently identifying the initial viscosity, particularly when the protein sample undergoes shear thinning. By defining the *T*_D_ as the temperature at which the maximum rate of viscosity decrease occurs the identification of this temperature becomes less subjective. The maximum rate of viscosity decrease was determined from the slope of the torque-temperature data as shown in Figure 2.

### 2.3. Effects of Heating Rate on Thermal Denaturation of Fish Collagens

Hoki and ling skin collagens denatured at a lower temperature with a slower heating rate. The calculated denaturation temperatures are shown in Table 1.

The effect of heating rate on the denaturation temperature observed for hoki collagen and ling collagen (Table 1) can be understood in the light of the model proposed by Miles and coworkers [7]. That is, that collagen thermal denaturation is an irreversible rate process, in which denaturation is a consequence of the total heat energy input and is dependent on the heating rate. This pattern of denaturation was observed with the hoki and ling skin collagens. Our results reported here support the proposal that collagen denaturation is an irreversible rate process.

While some triple helical character can form on cooling this is not re-alignment of the native triple helical molecule. Rather, this results from partial helix associations among random chains, which occurs as part of the gelation process. The *C*-terminal pro-collagen proteins rely on a complex set of interactions to establish chain recognition and alignment [10]. Thus, it is not surprising that the released chains from unraveled helices do not re-align without these pro-collagen portions of the α chains.

**Figure 3:**
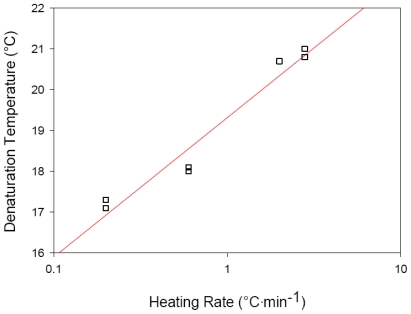
Logarithmic relationship between RVA-determined denaturation temperature and heating rate for hoki collagen (*R*^2^ = 0.954).

The collagen denaturation temperature increases logarithmically with heating rate [6,7,9]. The hoki collagen *T*_D_ also appeared to increase logarithmically with heating rate (Figure 3). This relationship reflects the different temperature-time histories undergone by collagens heated at different rates. For example, collagen heated from 10 to 28 °C at 0.2 °C/min is subjected to temperatures above 10 °C for 10-times as long as when a heating rate of 2 °C/min is used. Since collagen denaturation is a kinetic process, collagen subjected to longer periods of increased temperatures (e.g., when heated at 0.2 °C/min) results in the collagen denaturing at lower temperatures than when a higher heating rate is applied. Because *T*_D_ scales logarithmically with the heating rate, determination of the equilibrium melting temperature by extrapolating the heating rate data back to zero becomes impossible. This is because either collagen denaturation is a non-equilibrium process, as argued by Miles and colleagues [7,9], and therefore the concept of an equilibrium temperature is meaningless, or the equilibrium temperature is lower than previously thought and lower than that attainable in heating rate experiments, as suggested by Leikina and coworkers [6].

### 2.4. Effects of Solvents on Denaturation Temperature

The overall stability of the collagen molecule is defined by the total of hydrophobic, ionic, and hydrogen bonds and the water structure within and around the molecules. Introducing reagents that perturb the balance of these bonds and noting their effects on molecular stability helps us to understand the nature of the collagen molecule [11–14]. We determined the effects of low concentrations of urea on the thermal denaturation properties of these fish collagens. As shown in Table 2, the *T*_D_ of hoki and ling skin collagens decreased with increasing concentrations of urea (0.1–1 M). These results are similar to those of Usha and Ramasami, who studied rat tail tendon collagen [14]. They explained the effect of urea as indicating the importance of hydrophobic interactions in the stability of collagen. They proposed that urea disrupts the hydrogen bonding of the highly organized water in the collagen structure, and therefore competes with the hydrophobic forces.

In collagen research, acetic acid at concentrations of 0.1–0.5 M is a traditional solvent for swelling and solubilizing collagen fibrous structures with eventual release of monomers if acid-labile crosslinks are present. However, acetic acid is seldom used in manufacturing because of its strong odor, and citric acid is used instead. We investigated the effect of 0.1 M citric acid as well as different concentrations of acetic acid on the thermal denaturation properties of fish collagens. As shown in Table 2, *T*_D_ decreased as the concentration of acetic acid increased from 0.1 to 0.5 M. In addition, the effect of 0.1 M citric acid on the *T*_D_ of hoki and ling collagens was similar to that of 0.5 M acetic acid.

At high concentrations (4–6 M), urea denatures proteins by disrupting hydrogen bonds. Studies on collagen from the leather industry have shown that urea has a lyotropic effect on collagen, whereby the oxygen atom of the urea molecule binds to the amino group of the peptide linkage. This opens the structure of the molecule as the salt linkages are disrupted. Subsequently, the salt linkages bind hydrogen and hydroxyl ions leading to increased hydration of the collagen molecule. The overall result is swelling of the collagen, and ultimately, denaturation [15]. Acetic acid interacts in a similar manner, disrupting the hydrogen bonding across the peptide bond causing swelling of fibers and denaturation. This is particularly notable at high concentrations (>1 M). However, as noted above, acetic acid is traditionally used at concentrations from 0.1 to 0.5 M in collagen studies.

The triple helical structure of collagen has a single interstrand N-H _Gly_.....O=C _Xaa_ hydrogen bond per triplet [16,17]. This hydrogen bond stabilizes the collagen monomers. In addition, hydroxyproline has a significant role in stabilizing the collagen molecule [18]. The concept of the formation of water bridges from the hydroxyl group of the hydroxyproline residue to carbonyl groups to stabilize the helix was discussed by several workers in the 1970s [19,20]. In hydrated collagen, water that is bound in the form of water of hydration accounts for approximately 20% of the weight of the protein [21]. Detailed analyses of the hydration structure of a collagen-like peptide indicated repetitive patterns of water bridges, which were proposed by Bella and colleagues to stabilize the collagen triple helix [22]. The ordering of water around the collagen triple helices by extensive hydrogen bonding indicated that denaturing of collagen triple helices would involve an entropy increase not only due to the macromolecule, but also to water. The water bridge model was supported by the findings of other workers during stability studies of synthetic collagen-like polymers with increasing hydroxyproline content [9]. However, Shoulders and Raines [23] disagreed with this finding. Instead, they proposed that hydroxyproline stabilizes the helix via stereoelectronic forces, and that water bridges cannot be fundamentally important for triple helix stability. Their work was similarly based on the development of collagen-related peptides and examination of their thermal stability.

Whether hydroxyproline exerts it influence by supporting water bridges or by some other mechanism, it is clear that collagen triple helix thermal stability is highly dependent on hydrogen bonding. This is supported by the effects of increasing concentrations of urea and acetic acid on the thermal denaturation temperatures of fish collagens found in this work (Table 2). These reagents exert their effects on collagen by disrupting hydrogen bonding. That these effects occur even at the low concentrations investigated has significant implications for processing of marine collagens. While hoki and ling are both cold-water species commonly found at similar depths (200–800 m), their skins differ considerably in thickness and their collagens contain different proportions of hydroxyproline. The hydroxyproline content of hoki and ling skin collagens were determined as 50 and 42 aa/1000 amino acid residues, respectively (compared with 94 aa/1000 in collagen from calf [2]). The different properties of collagens extracted from various fish species are likely to result from crosslinking profiles (relevant to preparation methods) and the amount of imino acids, particularly hydroxyproline. The hydroxyproline content has a significant influence on the thermal stability as previously reported [2,18] and as illustrated in this work. Other properties resulting from the subtle differences in amino acid make-up among collagens from various species will become apparent as more species are investigated.

The debate over whether collagen denaturation is an equilibrium or non-equilibrium kinetic process has a direct bearing on the industrial uses of collagen. Whether collagen denaturation is a non-equilibrium process or not, it has a very low equilibrium temperature above which collagen is unstable. This means that when hydrated collagen is exposed to elevated temperatures even for short periods it will start to denature. If the time/temperature dependence of collagen thermal denaturation is understood in terms of irreversible rate kinetics this indicates that if the temperature is raised at any stage during processing irreversible changes will occur in the population of molecules being extracted. These changes cannot be reversed by simply lowering the temperature, and that product will retain the modified character that it achieved during the period of increased temperature. This will also apply to any collagen product during storage. Similarly, the type of solvent used during manufacture will significantly affect the thermal stability of the collagen. These results are especially important if the objective is to make a collagen product that relies on the native triple helical configuration for its functional properties. While this imposes restrictions on such a product, the results clearly define the conditions required to formulate and store a collagen product retaining triple helical structure. The low denaturation temperature of fish collagens can be an advantage for manufacturing, *i.e*., in general, low-temperature processing is less expensive than high-temperature processing. New sources of collagens with properties that differ from those of other, commercially available collagens provide new opportunities for industries that produce collagen products.

## 3. Experimental Section

### 3.1. Hoki Skin Collagen Preparation

Hoki skins were scraped clean with a knife to remove adhering muscle tissue, fat, and grey coloration and thoroughly washed in running tap water. Clean skins were mixed with 0.5 M acetic acid (1:40, w/v) and left stirring slowly for approximately 64 h at 7.5 °C. The mixture was centrifuged at 18,480× *g* for 50 min at 5 °C and the supernatant collected. To precipitate the protein, NaCl (30%) solution was added to the supernatant to give a final concentration of 1.0 M NaCl. After standing for 1 h, the salt-precipitated pellet of collagen protein was recovered by centrifugation at 18,480× *g* for 50 min at 5 °C. The pellet was redissolved in acetic acid (0.5 M) at 7.5 °C and then dialyzed to remove salt prior to freeze-drying.

### 3.2. Ling Skin Collagen Preparation

Ling skins were cleaned of all adhering tissue and stored at −40 °C until use. The skins were thawed overnight at 7.5 ± 0.5 °C and cleaned in running tap water for 10 min prior to washing in 0.05 M Tris-HCl pH 7.5 containing 1.0 M NaCl for 2 h. Skins were rinsed with water, immersed in acetic acid (0.5 M), and left to swell for approximately 64 h. The acid soluble collagen was recovered by centrifugation at 18,480× *g* for 50 min at 5 °C. The supernatant was freeze-dried immediately.

### 3.3. Sodium Dodecyl Sulphate Polyacrylamide Gel Electrophoresis (SDS-PAGE)

Collagens were separated by SDS-PAGE on 7.5% acrylamide gels (Criterion, BioRad, Hercules, CA, USA) according to the discontinuous gel method of Laemmli [24]. Sample and running buffers used for electrophoresis were as described by the manufacturer. Proteins were visualized with BioSafe™ Coomassie stain (BioRad, Hercules, CA, USA), and gels were photographed using a Syngene Genius Bio Imaging system.

### 3.4. Analysis of Hydroxyproline Content

Samples were analyzed at the Protein Microchemistry Facility, University of Otago, Dunedin, New Zealand. The samples were weighed in hydrolysis vials and hydrolyzed *in vacuo* for 1 h at 160 °C in 6 M HCl with 0.25% phenol. The resulting amino acid mixture was reacted with the fluorescent reagent phenyl isothiocyanate (PITC) before analysis on a reverse phase high performance liquid chromatography (HPLC) column [25].

### 3.5. Preparation of Gels for Analysis on the Rapid Visco™ Analyzer (RVA)

Concentrated 2.5% (w/v) collagen gels were prepared in a series of solvents, as follows: 0.1 M and 0.5 M acetic acid; 0.1 M citric acid; and 0.1 M, 0.5 M, and 1.0 M urea. Collagen samples were mixed with each solvent and were allowed to dissolve for approximately 20 h at 7.5 ± 1 °C prior to the first analysis. Hydrated collagen samples were stored on ice until RVA analysis when sample aliquots (30 g) were weighed out. Analyses were carried out over 2 days.

### 3.6. Determination of Viscosity with the Rapid Visco™ Analyzer

A Rapid Visco™ Analyzer (RVA) Series 4 (Newport Scientific Pty. Ltd., Warriewood, NSW, Australia) cooking, stirring viscometer was used to measure the viscosity of the collagen samples during heating from 10 to 28 °C. A Grant GR150 (Grant Instruments (Cambridge) Ltd., Cambridgeshire, UK) refrigerated water bath supplied cooling water at 10 °C to the RVA. The RVA was calibrated such that a single RVA viscosity unit corresponded to 12 cP (or 12 mPa·s) as per the manufacturer’s procedures.

Collagen samples (30 g) were weighed into chilled RVA aluminum sample canisters immediately before measurement. The samples were held at 10 °C for 2 min before the heating program was initiated. The samples were stirred at 60 rpm during heating from 10 to 28 °C at one of four heating rates (0.2, 0.6, 2, or 2.8 °C/min) and the torque on the mixing paddle recorded. The denaturation temperature was defined as the temperature at which the change in torque (viscosity) was greatest. To achieve this, torque (viscosity) data was smoothed with a Lowess filter (SigmaPlot 2002, SPSS (UK) Ltd., Surrey, UK) to remove the extraneous effects of signal noise. The filtered torque data was analyzed using a macro written in Excel Visual Basic for Applications (Microsoft Corp., Seattle, WA, USA) that identified the point at which the decrease in viscosity was greatest as determined from the minima in the slope of the torque-temperature data. Each point in the RVA torque slope curve was calculated using a regression slope equation that operated over a time region equivalent to 2 or 5% of the total experimental analysis time. The regression slope formula was as follows:

$$m=\frac{n\sum \left(xy\right)-\sum x\sum y}{n\sum \left(x^{2}\right)-{\left(\sum x\right)}^{2}}$$

where *m* is the slope at time *t*, *y* is the torque at time *t*, *x* is the temperature at time *t*, and *n* is the number of data points used to determine the slope at time *t*. Since the duration of the experiments varied with heating rate and the RVA data sampling rate was fixed at 0.5 Hz, *n* was set at 2% of the experiment duration for rapid heating rates (2 and 2.8 °C/min) and 5% for the remaining heating rates.

## 4. Conclusions

In this study, we determined the effects of heating rate and solvents on the TD of collagen extracted from skins of two fishes, hoki and ling. Solvents that disrupt hydrogen bonding decreased the TD of collagen, as did slower heating rates. These findings have implications for processing and storage of collagen products, as denaturation of the collagen molecule irreversibly changes its structure, which affects its properties as an ingredient.

## Figures and Tables

**Table 1:** Denaturation temperatures (*T*_D_) of hoki and ling skin collagens measured in 0.5 M acetic acid at different heating rates.

Collagen source	Heating rate (°C/min)	Denaturation temperature (*T*_D_) °C
Hoki *(Macruronus novaezelandiae)*	0.2	17.2
	0.6	18.5
	2.0	20.7 *
	2.8	20.9
Ling *(Genypterus blacodes)*	0.6	16.2 *
	2.0	17.5

Note: Data are means of duplicates, except points marked by an asterisk, which are single measurements.

**Table 2:** Effects of solvents on *T*_D_ of hoki and ling collagens.

Solvent	Concn. (M)	*T*_D_ Hoki	pH	*T*_D_ Ling	pH
Urea	0.1	23.1	3.7	19.7	4.6
Urea	0.5	21.7	3.7	18.7	4.6
Urea	1	20.0	3.9	17.3	4.7
Acetic acid	0.1	22.2	3.5	19.4	3.5
Acetic acid	0.5	20.7 *	3.0	17.5	3.0
Citric acid	0.1	20.6	2.3	17.7	2.4

Note: Data are means of duplicates, except point marked by an asterisk (single measurement).
